# An Agent-Based Model of the Stellar Mass Distribution

**DOI:** 10.3390/e28080895

**Published:** 2026-08-10

**Authors:** Enrique Raúl Olguín-Rodríguez, Carlos Gershenson, Enrique Vázquez-Semadeni

**Affiliations:** 1 Instituto de Investigaciones en Matemáticas Aplicadas y en Sistemas, Universidad Nacional Autónoma México, Ciudad de México 04510, Mexico; enriraul.rdz@gmail.com; 2 School of Systems Science and Industrial Engineering, Binghamton University, Binghamton, NY 13902, USA; 3 Centro de Ciencias de la Complejidad, Universidad Nacional Autónoma México, Ciudad de México 04510, Mexico; 4 Instituto de Radioastronomía y Astrofísica, Universidad Nacional Autónoma México, Morelia 58089, Mexico; e.vazquez@irya.unam.mx

**Keywords:** agent-based modeling, initial mass function, competitive accretion, stellar mass distribution

## Abstract

The Initial Mass Function (IMF) describes the distribution of stellar masses formed in a stellar cluster and constitutes a fundamental constraint for theories of star formation. In this work, we investigate the emergence of the IMF slope using a minimal agent-based model inspired by preferential attachment mechanisms. The model represents stars as accretion centers embedded in a reservoir of infalling material, where mass growth occurs through competitive accretion at a rate proportional to a tunable power of the stellar mass, and where fragmentation of accretion centers is allowed. We systematically explore the effects of accretion and fragmentation probabilities on the resulting mass distribution. The simulations show that competitive accretion alone leads to the dominance of a single massive object, whereas the inclusion of fragmentation regulates mass growth and produces a stable mass spectrum. For a specific range of accretion exponents, the resulting stellar mass function exhibits a logarithmic slope close to the Salpeter value. These results indicate that the interplay between fragmentation and mass-dependent accretion can produce a power-law slope comparable to the observed IMF under the simplified assumptions of the proposed agent-based model.

## 1. Introduction

The initial mass function (IMF) is a fundamental concept in astrophysics, describing the stellar mass distribution of a newly formed stellar population [1,2,3,4,5]. Roughly speaking, this function tells us how many stars form in a given star formation episode in a certain region in the interstellar medium of galaxies. The IMF plays a crucial role in understanding the origin of the stellar population of galaxies, their evolution, and the overall distribution of mass in the universe. Numerous models have been proposed to explain the origin of the IMF. Reference [6] groups these models into four main classes according to the physical processes assumed to generate IMF. According to them, the IMF may originate from fragmentation, whether it be turbulent fragmentation [7,8,9,10,11,12,13,14], gravitational fragmentation [15,16,17,18] or domain packing [19] with the fragmentation subject to an opacity limit, which sets a minimum stellar mass [20,21,22,23,24,25,26,27]. Alternatively, it may originate from feedback processes [28,29,30], competitive accretion, or between fragments [2,20,31,32,33,34,35,36,36], or it may be due to coalescence or collisional build-up [37,38,39,40]. In this work, our main objective is to investigate how various physical processes involved in star formation affect the resulting stellar mass distribution. Given the complexity of these processes and the computational cost of fully detailed simulations, we aim to develop a simplified, computationally inexpensive “toy model” that still captures the essential dynamics of star formation. To achieve this, we adopt an agent-based modeling approach, which enables us to explore different scenarios and perform multiple experiments efficiently. The structure of the paper is the following: Section 2 introduces star formation, describes the main characteristics of accretion competitive model, and discusses some relevant studies of the initial mass function. In Section 3, we present agent-based models and some other concepts used in our modeling. In Section 4, we present our model, its components, basic rules, and main characteristics. Next, in Section 5, we show the results, and in Section 6, we draw our main conclusions.

## 2. Star Formation

The process of star formation originates from the gravitational collapse of a molecular cloud or parts thereof. Molecular clouds have a highly non-homogeneous structure, with significant density fluctuations. Moreover, since denser gas has shorter free-fall times, molecular clouds collapse in a non-homologous manner; that is, the denser material completes its collapse to form singularities (protostar), while most of the cloud is still falling at finite densities. The main characteristics of the competitive accretion model for the generation of the stellar IMF are as follows:

1. The densest regions within molecular clouds, known as molecular cloud *cores*, are in the process of gravitational contraction, and therefore develop a gravitationally-driven *collapse flow*. Since they are in general not uniform nor spherical, they develop multiple collapse centers (i.e., they *fragment*), onto which the lower-density material from the core continues to accrete, e.g., [41,42,43,44].
2. The collapse centers constitute the *protostars*, which grow in mass as they continue to accrete gas from the core. The latter, in turn, constitutes a *mass reservoir*. As a protostar’s mass increases, so does its gravitational pull, increasing the accretion rate onto it.
3. The mass distribution of the set of protostars arises as a consequence of the competition among them to accrete the available mass in the reservoir.
4. Accretion onto the fragments ends when the fragments reach sufficiently high densities, so that the gas becomes optically thick and begins to trap the heat generated by collapse-induced compression. This raises the internal temperature, until finally high enough densities and temperatures (of 150 g cm^−3^ and $1.5\times 10^{7}$ K, respectively) are reached that nuclear reactions (fusion) are initiated in their centers, “igniting” the star and disrupting the accretion flow. However, until this occurs, the protostars continue to draw gas from their environment, increasing their mass to a certain final value.

### 2.1. Initial Mass Function

One of the most fundamental unsolved problems in astronomy is the origin of the distribution of stellar masses, known as the *initial mass function (IMF)*. The IMF is an empirical distribution that describes the masses of stars at the time of their formation.

The IMF is fundamental in astrophysics because it determines the relative number of stars formed in different mass ranges within a star-forming region. Consequently, it plays a key role in the following:

- The chemical enrichment of galaxies, since massive stars produce and disperse heavy elements;
- The evolution of galaxies, determining the relative abundance of stars of different masses, which influences their luminosity, colour, and chemical evolution;
- The stellar feedback injected into the interstellar medium through radiation, stellar winds, and supernova explosions.

**Figure 1:**
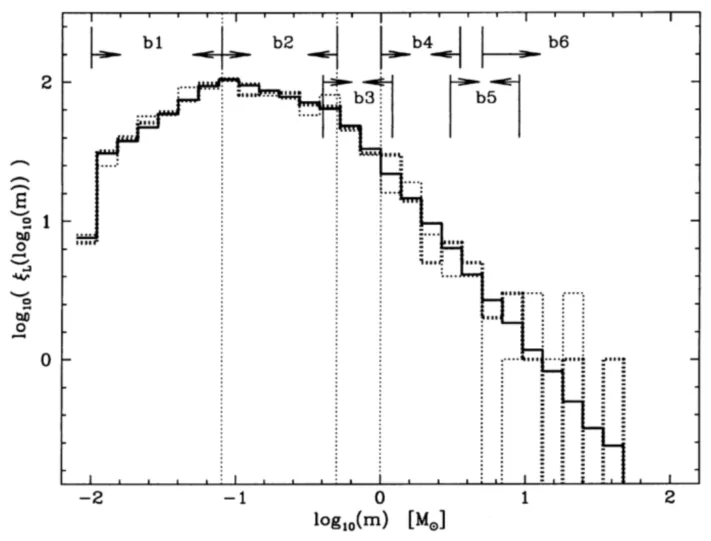
Initial mass function presented in Figure 2 of [3], where different reported IMF variations are compared and studied. The horizontal axis represents the stellar mass in logarithmic solar-mass units, $\log_{10}\left(m\right),\left[M_{⊙}\right]$, while the vertical axis shows the logarithmic stellar number distribution, $\log_{10}\left(\xi_{L}\left(\log_{10}\left(m\right)\right)\right)$. The regions labeled b1–b6 indicate different mass intervals used to fit power-law functions and analyze the IMF behavior across distinct stellar-mass regimes.

In the rest of this paper we will mostly be concerned with the slope of the high-mass end of the IMF. Ref. [1] was the first to propose a quantified initial mass function, which he called “*The original mass function*”. The author observed the luminosity function for main sequence stars in the solar neighborhood (by “main sequence stars”, we refer to the stars that are in the longest-lasting and most stable phase of their life cycle, where they fuse hydrogen into helium in their cores. This process releases energy, allowing equilibrium with the gravitational force that attempts to compress the star). By collecting and organizing the observational data of the distribution of stellar luminosities available at the time, he determined that the stellar mass distribution at high masses has the approximate form

(1) $$dN\left(M\right)\propto M^{-2.35}dM$$

where *M* is the stellar mass and N(M) is the number of stars in the mass interval (M,M+dM). One of the currently accepted forms of the IMF is a piece-wise power law. In particular, ref. [3] described it as illustrated in Figure 1.

Even though there are many ways to represent the IMF, in general the high-mass part is generally reported as a power law with the Salpeter slope. In the same way, there are a large number of different theoretical explanations for the origin of IMF. Nevertheless, the process known as *competitive accretion* [45] is one of the most widely accepted possible mechanisms responsible for its appearance.

In the competitive accretion scenario proposed by Bonnell et al. [46], protostars form with similar initial masses within a common gravitational potential. As the cluster evolves, stars located in denser, central regions gain preferential access to the available gas, accreting mass at higher rates than those in the outskirts. This process naturally leads to mass segregation and to the formation of massive stars in the cluster center. A fundamental ingredient in the assembly process of a star is the accretion rate of each protostar. Based on the classical Bondi–Hoyle–Lyttleton [47] accretion, Zinnecker [32] presents the accretion rate as

(2) $$\dot{M}\approx \alpha M^{2},$$

where α is a constant. This equation indicates that the accretion rate of a protostar is proportional to the square of its mass. On the basis of this accretion model, and using a kinetic equation approach for the distribution function, Zinnecker [32] concluded that the mass spectrum should have the form $dN\left(M\right)\propto M^{-1}d\log M$. In logarithmic mass intervals, this prediction corresponds to a slope of −2 in linear bins, as in Equation (1), close to the Salpeter value.

**Figure 2:**
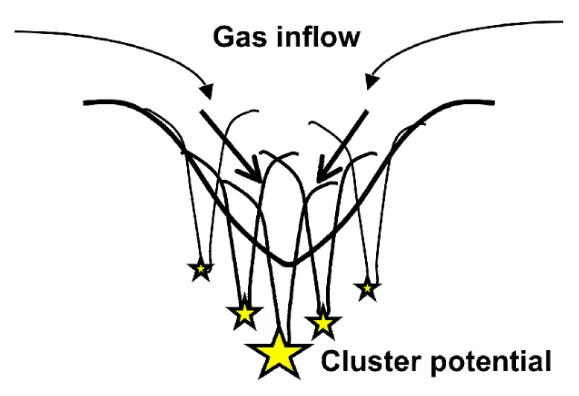
Schematic diagram of competitive accretion in a stellar cluster (adapted from Figure 7 [46]). The self-gravity of the gaseous dense core drives an infall flow and a fragmentation process that leads to the formation of *protostars*, which then continue to accrete at a rate proportional to their own masses and to the local inflow density. This is a highly chaotic process, in which random fluctuations in the accretion flow and the chaotic motions of the protostars lead to a competition for the infalling material, and numerical simulations show that it leads to realistic IMFs. This is the physical process that motivates the simplified agent-based model proposed in this work.

### 2.2. Jeans Length and Mass

The Jeans length and mass are fundamental concepts in astrophysics that define the minimum size and mass that a cloud of interstellar gas must have in order for gravity to overcome thermal pressure and allow gravitational collapse. In this paper we will consider them at the time of choosing parameters for our agent-based model for the IMF. The Jeans length, $L_{J}$, is derived from a linear instability analysis of an infinite and uniform gaseous medium [48], and it represents the critical wavelength separating perturbations that are stabilized by thermal pressure from those that become gravitationally unstable and undergo collapse. It is given by

(3) $$L_{J}=\sqrt{\left(\frac{\pi c_{s}^{2}}{G\rho }\right)},$$

where ρ is the density of the gas, $c_{s}$ is its sound speed, and *G* is Newton’s gravitational constant. The Jeans mass, $M_{J}$, is then often defined as the mass of a uniform sphere in this medium with radius equal to $L_{J}/2$, and is thus given by

(4) $$M_{J}=\frac{\pi^{5/2}}{6}\frac{c_{s}^{3}}{G^{3/2}\rho^{1/2}},$$

representing the maximum mass in a uniform medium that can be supported by the thermal pressure gradient against its self-gravity.

Although the Jeans mass and Jeans length are not explicitly incorporated into the model equations, they provide the physical motivation for including fragmentation as one of the fundamental processes considered in this work. In particular, these quantities illustrate that self-gravitating structures may become unstable and fragment into smaller condensations as their masses grow much larger than their Jeans mass through accretion, motivating the introduction of a fragmentation mechanism within the agent-based framework. A more detailed discussion of this connection is provided in Appendix B.

## 3. Agent-Based Model

### 3.1. Agent-Based Modeling

The term “model” is defined as “an abstract and simplified representation of given reality, either already existing or just planned;”. Models are commonly defined in order to study and explain observed phenomena or to foresee future phenomena [49].

Agent-based models (ABMs) constitute a technique that complements traditional analytical methods. In ABMs, a system is modeled as a collection of autonomous entities called agents, where an *agent* is a unit capable of making decisions based on a set of rules.

In contrast to analytical methods, which focus on modeling and characterizing the equilibrium of a system (“top-down” approach), the ABM offers the possibility of generating that equilibrium [50].

In this sense, ABMs have a bottom-up approach that provides a natural environment for studying complex adaptive systems, where individual interactions lead to emergent behavior [51,52], and is flexible, particularly for the development of spatial models, as it allows for the explicit representation of spatial heterogeneity and localized interactions between agents. Unlike differential equations, which often assume continuous and homogeneous distributions, ABMs can incorporate discrete and dynamic spatial structures, making them especially useful for modeling urban growth, land use changes, and the spread of epidemics, among many others [53].

In the specific case of our agent-based model, we can state that the rules governing agent behavior are inspired by the Barabási–Albert network growth model [54,55]. It is for this reason that it is necessary to understand the basic concepts and components that comprise the Barabási–Albert model, which we will address in the following section.

### 3.2. What Is an Agent?

There is no universal agreement on the precise definition of the term “agent”. Still, definitions tend to agree on more points than they disagree [56]. Agent characteristics are difficult to extract from the literature in a consistent and concise manner, because they are applied differently within disciplines [53].

In a very general way, an agent is a description of an entity that *acts* on its environment [57].

From a pragmatic modeling standpoint, several features are common to most agents ([58], extended and explained further by [59,60,61,62]).

- **Autonomy:** Agents are autonomous units. They can process information and interact with other agents to make independent decisions.
- **Heterogeneity:** Agents can have different capabilities, knowledge, and objectives, allowing the development of autonomous individuals with distinct behaviors within a system.
- **Active:** Agents are active because they exert independent influence in a simulation.
- **Reactive:** Agents can be designed to sense of their surroundings and act accordingly.
- **Interactive:** Agents have the ability to communicate with other agents.
- **Mobility:** The movement of agents is a particularly useful feature, not least for spatial simulations.
- **Adaptation/Learning:** Agents can be designed to be adaptive. They can alter their state depending on the situation.

In this work, we define an agent as *a unit that possesses a set of skills and characteristics that are governed by a set of simple rules. Agents are capable of interacting with other agents and their environment.* Although these characteristics are commonly used in the general agent-based modeling literature, they should not be interpreted literally in the present work. The agents considered here represent abstract accretion centers governed by externally defined probabilistic rules and do not possess cognition, learning capabilities, or autonomous decision-making processes.

### 3.3. Barabási–Albert Model

**Figure 3:**
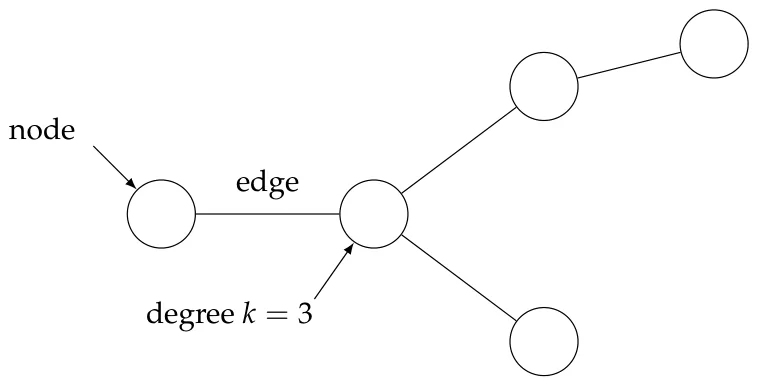
Example of a network. Circles represent nodes and lines represent edges (connections, or links). The central node has degree k=3, indicating that it is connected to three neighboring nodes.

Barabási and Albert addressed the origin of the power-law degree distribution observed in networks [54,55]. A network (or graph) is a mathematical structure composed of nodes (vertices) connected by edges (links) (Figure 3). In this representation, nodes correspond to the entities of the system, whereas edges represent interactions or relationships between pairs of entities. Since different nodes may be connected to a different number of neighbors, it is useful to characterize each node by its *degree*. The degree $k_{i}$ of node *i* is defined as the number of edges connected to that node. In the Barabási–Albert model, the probability that a newly added node connects to an existing node increases with its degree. Consequently, nodes with larger degree have a higher probability of receiving new connections.

Barabási and Albert studied many real networks and concluded that they exhibit a scale-free nature as a consequence of mainly two processes: *Growth* and *Preferential attachment*. Those authors then constructed the so-called Barabási–Albert model, consisting of the following:

1. *Growth:* The system is represented as a network (graph) whose nodes represent individual agents, and whose edges represent interactions (or connections) between pairs of agents. The model starts with a small number of nodes $m_{0}$. At each time step, a new node is added and connected by *m* edges to *m* different nodes already present in the network.
2. *Preferential Attachment*: The selection of nodes to which the new node connects is determined by a probabilistic function that depends on the degree $k_{i}$ of node *i*, defined as the number of connections of that node:
  (5) $$\Pi \left(k_{i}\right)=\frac{k_{i}}{\sum_{j}k_{j}}.$$

These simple rules imply that new nodes will connect to highly connected nodes with a higher probability, making them even more highly connected. Numerical simulations show that this kind of network has a power-law degree distribution with exponent $\gamma_{BA}\approx 3$, meaning that the probability of a node having degree *k* follows a power-law function $P\left(k\right)\approx k^{-\gamma }$. This implies that most nodes have low degree, but a few nodes have a very high degree.

In the present work, we use this preferential attachment mechanism as an analogy for competitive accretion. Instead of describing the growth of node connectivity, we assume that more massive accretion centers have a higher probability of acquiring additional mass. This analogy provides the basis for the accretion rule introduced in Equation (6).

## 4. Description of the Agent-Based IMF Model

The main objective of the model we propose is to investigate whether the combined action of accretion and fragmentation can generate a stellar mass distribution that can be described by a power law and exhibits a slope consistent with that observed by Salpeter.

Before describing the model rules, it is important to clarify the interpretation of the agents employed in the simulations. Throughout this work, the agents represent *abstract accretion centres* rather than individual stars in a strict physical sense. These centres correspond to localized concentrations of mass that compete for a finite mass reservoir and may undergo fragmentation according to the model rules. From a physical perspective, they may be viewed as idealized analogues of dense molecular cores or young protostellar objects; however, no direct one-to-one correspondence with any specific astrophysical entity is assumed. This level of abstraction allows the model to focus on the collective effects of accretion and fragmentation without explicitly modeling the internal structure of molecular clouds or the detailed hydrodynamics of star formation.

We consider a non-spatial model, where agents have no specified position and interact only through their competition for a common reservoir of accretable material. Initially, the system contains a population of accretion centers that can accrete infalling material, competing with the rest of the population for the available mass. Thus, our model represents the process of competitive accretion [2]. The agents are governed by two main processes, namely *accretion* and *fragmentation*, which are described below.

### 4.1. Accretion

To reproduce the mechanism of competitive accretion, we were inspired by the Barabási–Albert model, in particular by *preferential attachment* (Section 3.3). We consider a modification of the expression of preferential attachment (Equation (5)) where we make an analogy between the node’s degree and an agent’s mass, because the probability of an agent accreting mass increases as its own mass increases, according to the Bondi–Hoyle–Lyttleton accretion model [47]. Thus, we replace the node’s degree $k_{i}$ with the agent’s mass, $m_{i}$, raised to some power α. Hence, the accretion probability of an agent is given by

(6) $$\Pi \left(m_{i}\right)=\frac{{m_{i}}^{\alpha }}{\sum_{j}{m_{j}}^{\alpha }},$$

where $m_{i}$ is the mass of an agent *i* and α is a constant. We can consider Equation (6) as an analogy to the accretion rate.

### 4.2. Fragmentation

In addition to accretion, the model considers another process that largely determines the behavior of the agents’ mass distribution, namely, fragmentation. This process limits the growth of massive agents while simultaneously generating a larger population of lower-mass agents.

We propose that the probability of fragmentation increases with the mass of an agent, i.e., the greater the mass of an agent, the greater the probability that it will fragment. This assumption is qualitatively motivated by the idea that more massive objects are expected to contain a larger number of Jeans masses, each of which may become gravitationally unstable and collapse independently, leading to hierarchical fragmentation.

However, the fragmentation probability adopted here is a phenomenological prescription inspired by this physical picture rather than a direct implementation of Jeans fragmentation. Its purpose is to capture, in a simplified manner, the tendency of more massive accretion centers to fragment more readily within the context of the present toy model.

We therefore introduce the following fragmentation probability function:

(7) $$P\left(m_{i}\right)=1-m_{i}^{-\beta },$$

where $m_{i}$ is the mass of agent *i*, and β>0 is a tunable parameter controlling how rapidly the fragmentation probability increases with mass. In the present model, agent masses satisfy $m_{i}\geq 1$, which guarantees that $m_{i}^{-\beta }\leq 1$ and therefore that the fragmentation probability remains in the valid range

$$0\leq P(m_{i})<1.$$

**Figure 4:**
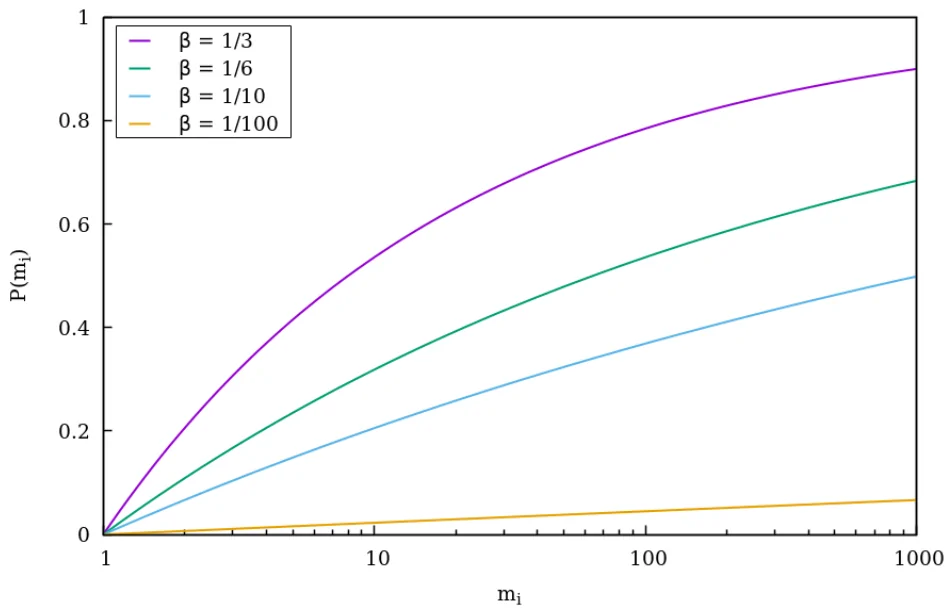
Fragmentation probability obtained from Equation (7) as a function of the mass $m_{i}$ for different values of the parameter β. Larger values of β lead to a faster increase in the fragmentation probability.

Thus, low-mass agents have a lower probability of fragmentation, whereas more massive agents are increasingly likely to fragment. The behavior of this function for different values of β is shown in Figure 4.

### 4.3. Algorithm

The model is initialized with a set of (*N*) agents (accretion centers), where each agent (i∈N) has an initial mass ($m_{i}=1$) and no spatial position. Unless otherwise stated, all simulations start with (N=1000) agents and a reservoir of *M* units of accretable mass. The choice of identical initial masses is intended to avoid introducing a predefined mass spectrum, allowing the resulting distribution to emerge solely from the stochastic accretion and fragmentation dynamics. Then, the following cycle is iterated:

1. A unit of accretable mass is added to the system (Note that, in this abstract model, mass is represented in arbitrary units and does not correspond to any specific physical mass scale. Nevertheless, in view of the mass distributions obtained from the model, a useful comparison is obtained by equating a model mass unit to ∼0.1 $M_{⊙}$, where $M_{⊙}$ is the mass of the Sun.).
2. The accretable mass is competed by the agents. Each agent has a probability of accreting the unit mass given by Equation (6).
3. The selected agent increases its mass by one unit, $m_{i}=m_{i}+1$.
4. Agents have a probability of fragmentation given by Equation (7). When an agent of mass ($m_{i}$) fragments, it is replaced by ($n=m_{i}$) agents of unit mass, which subsequently participate in the accretion process.

The simulation is terminated after the predefined reservoir of accretable mass has been exhausted. Although mass is introduced into the system one unit at a time, the total amount of accretable material is fixed at the beginning of each simulation. The algorithm is described in detail in Appendix A.

## 5. Simulations and Results

One of the main points of interest we have in this project is to determine which physical processes are necessary to obtain a mass distribution that can be described by the Salpeter slope. To do this, we will therefore study these processes separately and observe the distributions obtained. First, we vary the accretion velocity, which is described by the variable α. Next, we study the relevance of the fragmentation process by varying the fragmentation probability β and study the impact this has on the agent mass distribution.

### 5.1. Accretion Only

As we have seen, ref. [32] generated an analytical model for a number of “seeds” that grow into stars by accretion. With this in mind, the value of α in the accretion equation can be considered as the control parameter of our accretion rate. This is because the higher the value of α, the greater the probability that an agent can accrete.

**Figure 5:**
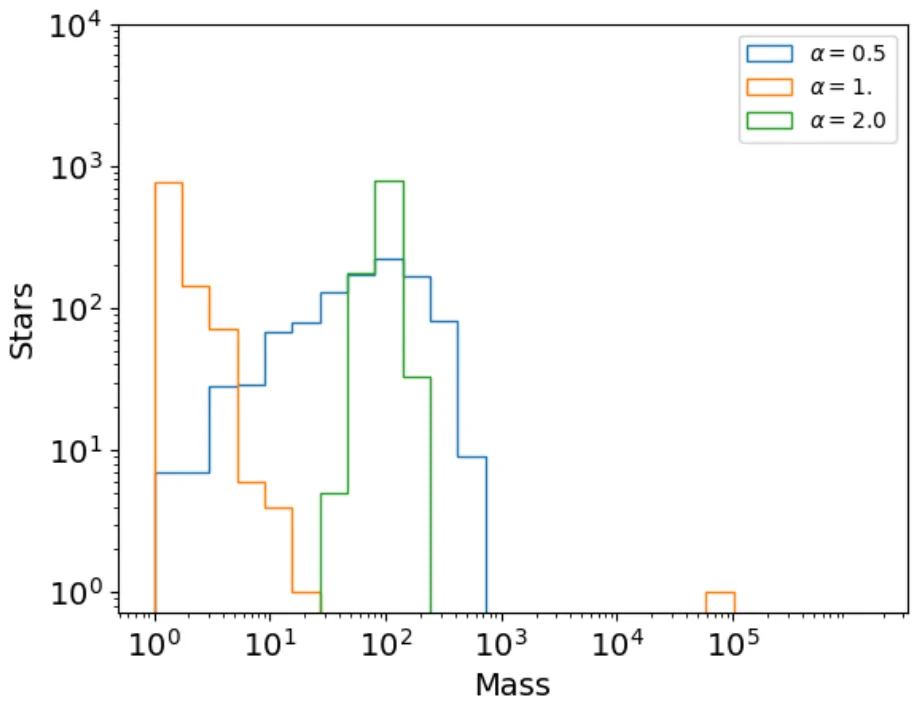
Histograms of the agent masses obtained from the accretion-only model for representative values of the accretion parameter α=0.5,1 and 2. These values illustrate the different mass-distribution regimes discussed in the text.

For this study, we will consider a constant number of agents (1000), providing a fixed amount of accretable material for each iteration in the model, until the total of all agent masses is $10^{5}$. Also, we will perform this process by varying the accretion parameter in the range α>0, to observe its effect on the agent mass distribution. Figure 5 shows three examples of the resulting distributions for the cases α=0.5,1, and 2.

For this range of values of α, we can observe three groups of cases as follows:

**Case** **1** (α∈(0,1)): Simulations within this range tend to have distributions that increase with agent mass. That is, the distribution has a positive slope.
**Case** **2** (α∈[1,2)): In this range, we observe the presence of a single accreting agent with a much larger mass compared to the rest of the distribution. It is worth mentioning that the larger α is, the greater the difference in mass between the massive object that accretes most of the accretable material and the rest of the agents. If we omit the presence of this supermassive object, the remaining distribution can be represented by a power law whose exponent is close to −3 with a negative slope.
**Case** **3** (α∈[2,∞)): The behavior we have observed in the agent mass distribution in this interval is confined to a narrow mass range. In addition, the higher the value of α, the narrower the mass range.

**Figure 6:**
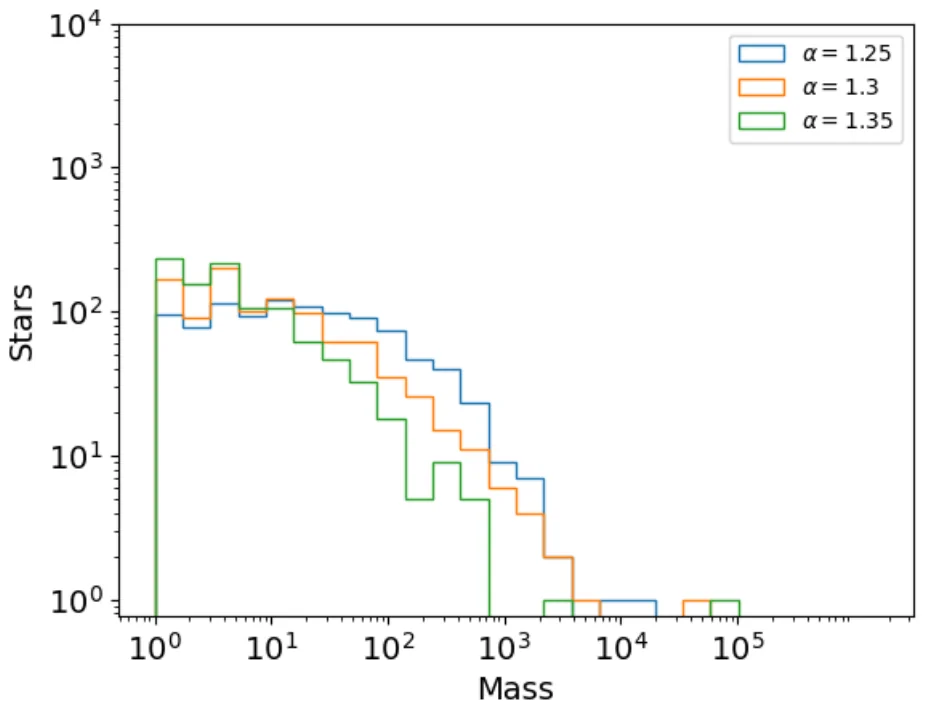
Agent mass distributions obtained for α=1.25,1.30 and 1.35. These values illustrate the onset of the separation between the most massive object and the rest of the agent population.

In view of these results, we have performed further tests with α in the (1, 2) interval, mainly to determine up to which values of α the mass of the most massive object is still not far from the rest of the distribution. In particular, Figure 6 shows that the mass of the most massive agent begins to separate from the rest of the distribution as α varies from ≈1.25 to ≈1.35.

### 5.2. Fragmentation Probability Analysis

In this section, we are not interested in modeling the outcome of fragmentation events, such as the generation of new bodies. Instead, we focus on determining the range of values of the parameter β that produces fragmentation frequencies compatible with sustained accretion.

One of the points of interest regarding the fragmentation process is to determine the range of β values for which the growth of the agents’ masses is allowed. If the probability of fragmentation is too high, fragmentation will be so frequent that it will prevent the mass growth of the accretion centers, whereas, if the probability is too low, in practice fragmentation will not occur.

**Figure 7:**
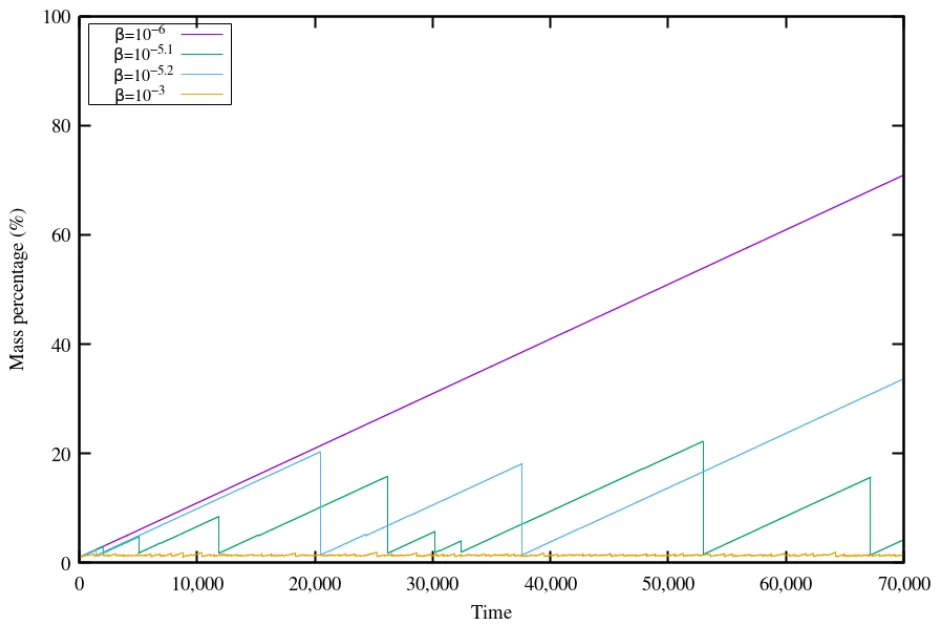
Evolution of the mass in the accretion centers (agents) shown as a percentage of the total mass in the system (agents plus accretable material) for various values of β, the fragmentation probability parameter in Equation (7). We observe that there are two values that delimit the fragmentation behavior: for $\beta =1\times 10^{-6}$, the probability is so small that fragmentation never happens, and for $\beta =1\times 10^{-3}$ the value is so large that the growth of the agents’ masses is prevented. The time axis is given in number of iterations of the model.

To determine this range we perform the following experiment: We consider a model that has a constant small population of *n* agents, and we add a constant amount of accretable mass *m*. Since in this case we are not interested in the details of the accretion process, for simplicity, we take α=1 and, after determining a winner, we calculate the probability for each of the agents to fragment. In the case that an accretion center does fragment, we reduce its mass by one unit and return all accretable material to *m*. Thus, the sum of the agent’s masses and the accretable mass set will always be constant. In Figure 7, we plot the sum of the agents’ masses as a percentage of the total existing mass (the sum of the accretable material plus the total mass of the agents) as a function of time for various values of the fragmentation parameter β.

Figure 7 shows only those results where the agents are able to accrete, including the lower limit of β where fragmentation never occurs during the entire number of iterations considered. Based on the results, we have determined that the useful range for the value of β is at $\beta \in [1\times 10^{-4},1\times 10^{-5}]$ for the number of iterations of the model we consider.

### 5.3. Accretion and Fragmentation

After studying the two main processes of our model separately, and determined the useful values of the parameters, we can consider them combined.

The values contemplated for the parameters are as follows:

- Accretion rate: α∈{1.25,1.3,1.35}.
- Fragmentation probability:
  $\beta \in \{1\times 10^{-4},1\times 10^{-5}\}$.

**Figure 8:**
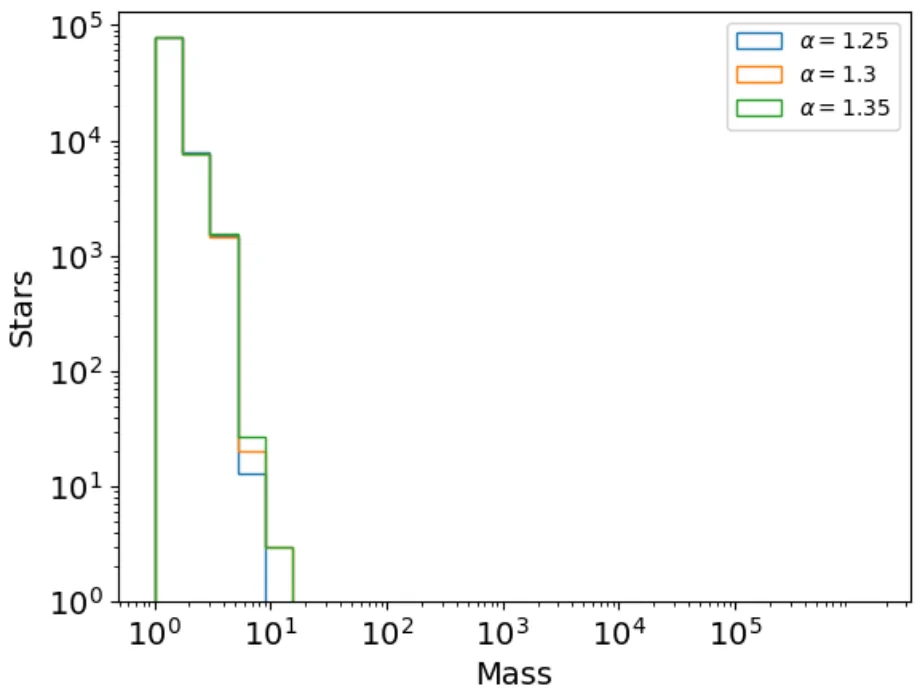
Agent mass distributions obtained by the agent-based model including both accretion and fragmentation, for various values of α and $\beta =1\times 10^{-4}$.

**Figure 9:**
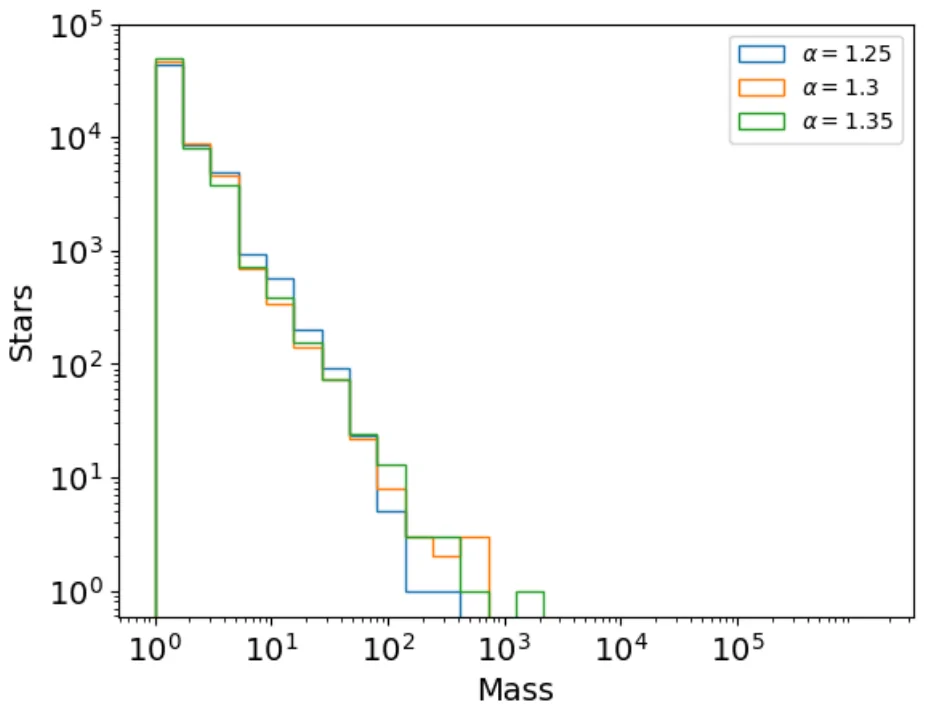
Agent mass distributions obtained by the agent-based model with accretion and fragmentation, for various values of α and $\beta =1\times 10^{-5}$.

The results obtained (shown in Figure 8 and Figure 9) highlight the importance of these two processes as necessary for reproducing the desired mass distribution. In both figures, there is a power law that can represent the distribution of stellar masses for each of the three values of α considered. For each value of β, we note the following:

**Case** $\beta =1\times 10^{-4}$ (Figure 8): The mass distributions are mainly concentrated in the low-mass region and have very steep slopes ≈−5.

**Case** $\beta =1\times 10^{-5}$ (Figure 9): In this case, the agent mass distributions have shallower slopes (≈−1.5), and therefore contain a larger number of high-mass agents. These values of the slope are closer to that found originally by Salpeter. It is worth mentioning that in these distributions, there is a similarity in the lower mass regions and small variations in the higher mass regions for different α values.

Given the large number of agents at low masses, we suggest that for this value of β the fragmentation process remains efficient enough to prevent the runaway growth of individual agents. From these results, we can tentatively conclude that β is the main parameter controlling the slope, indicating that the fragmentation process strongly influences the resulting mass distribution.

These results also suggest that the emergence of the mass distribution is governed by collective interactions among the agents, rather than by the independent evolution of individual accretion centres. This observation naturally invites comparison with other self-regulating approaches proposed in the literature.

The present agent-based framework shares conceptual similarities with other self-regulating approaches proposed to explain the origin of the stellar mass distribution. In particular, Weidner and Kroupa [63] identified a relation between the mass of the most massive star in an embedded cluster, $m_{max}$, and the total stellar mass of the cluster, $M_{ecl}$. This empirical relation motivated the development of the optimal sampling framework [64], in which stellar masses are constrained by the total mass available to the forming stellar population rather than being treated as independent stochastic realizations. More recently, Yan et al. [65] compared this relation with stochastic sampling models, whereas Gjergo et al. [66] showed that optimal sampling can naturally emerge from a variational, constraint-based analysis combined with fragmentation. Although our model differs substantially in its implementation, both approaches highlight the importance of collective regulation under a finite mass reservoir. Exploring the conceptual connections between these frameworks represents an interesting direction for future work.

## 6. Conclusions

In this work, we explored the variation in the stellar mass function using an agent-based model that incorporates accretion and fragmentation as its main mechanisms, inspired by the Barabási-Albert network model for growth through *preferential attachment*. By systematically varying the accretion and fragmentation probabilities, we analyzed their influence on the resulting mass distribution and on the slope of its high-mass tail.

The main conclusion that can be drawn from our results is that, within the assumptions of the present agent-based model, the combined action of accretion and fragmentation can generate a power-law agent mass distribution with a high-mass slope comparable to the Salpeter value. Models with accretion only can also generate power law mass distributions for accretion parameters (the exponent in the accretion law, Equation (6) α≳1.3, but with the additional formation of a very massive agent outside the range of the power-law distribution. The inclusion of a fragmentation prescription given by Equation (7) prevents the formation of such a super-massive object while preserving the general power-law mass distribution.

From the perspective of IMF research, the proposed model offers a flexible and conceptually transparent framework for investigating how simple accretion and fragmentation rules can give rise to collective mass distributions. Although the model is intentionally abstract and does not attempt to reproduce the detailed physics of star formation, it provides a computationally inexpensive environment for exploring qualitative behaviors and testing hypotheses regarding the emergence of IMF-like properties.

Nevertheless, this approach also presents clear trade-offs. While agent-based models sacrifice detailed physical realism, they gain simplicity, adaptability, and computational tractability. This makes them particularly suitable for studying the collective effects of simplified interaction rules and for identifying mechanisms that may contribute to the formation of stellar mass distributions, rather than for reproducing star-forming regions in full physical detail.

## Figures and Tables

**Figure A1:**
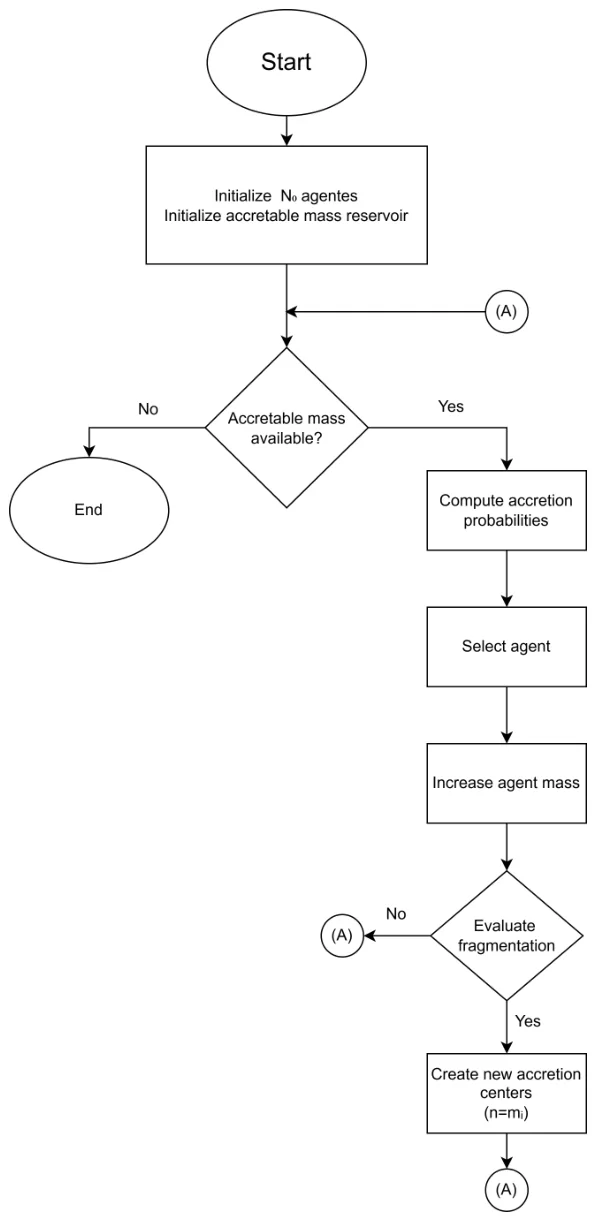
Flow diagram of the simulation cycle. After initialization, agents compete for accretable mass according to Equation (6). The selected agent may subsequently fragment according to Equation (7). The procedure is repeated until the accretable mass reservoir is exhausted.

## Data Availability

The data and code supporting the reported results are available at https://github.com/enriraul/An-Agent-Based-Model (accessed on 2 August 2026). The repository contains the simulation scripts, model parameters, and generated datasets used in this study.

## Appendix A. Detailed Model Implementation

### Appendix A.1. Initial Conditions and Model Setup

Here, we provide a detailed description of the model implementation.

The model consists of a population of agents representing abstract accretion centers. These agents are not intended to represent individual stars in a strict physical sense, but rather localized concentrations of mass that compete for available material and may undergo fragmentation according to the rules described in the main text. The use of abstract accretion centers allows the model to focus on the collective effects of accretion and fragmentation without introducing additional physical processes or spatial degrees of freedom.

At the beginning of each simulation, the system is initialized with $N_{0}$ accretion centers, or *agents*, each having a unit mass ($m_{i}=1$). The model intentionally starts from a homogeneous population of accretion centers. This choice removes any pre-existing mass hierarchy and allows the resulting mass spectrum to emerge exclusively from the interaction between accretion and fragmentation. Consequently, any mass hierarchy observed at later stages is generated by the model dynamics rather than imposed through the initial conditions.

Instead of modeling the gas distribution explicitly, all available material is represented by a common reservoir of accretable mass. The reservoir contains a finite amount of accretable mass specified at the beginning of each simulation. At each iteration, one unit of mass is extracted from this reservoir and becomes available for accretion according to the probabilistic rule described in Equation (6). In this way, agents compete for a shared mass resource, while the total amount of material available to the system remains finite. The initialization procedure is intentionally simple and is designed to minimize the influence of assumptions unrelated to the accretion and fragmentation mechanisms under study.

### Appendix A.2. Accretion Process

Once the system has been initialized, the growth of the accretion centers proceeds through a probabilistic accretion process. At each iteration, a single unit of mass becomes available for accretion and is assigned to one of the agents according to the preferential attachment probability rule given by Equation (6). This distribution is then used to perform a stochastic selection of a single agent, which then receives (“accretes”) the unit mass released from the reservoir. Because of the preferential attachment implied by Equation (6), agents with larger masses have a higher probability of being selected, while all agents retain a non-zero chance of accreting mass.

Agent selection is performed through weighted random sampling using the normalized probabilities by assigning each agent an interval of length equal to its probability along the unit interval in the real axis. A random number in the interval [0,1) is then generated, and the new mass is assigned to the agent whose interval contains the generated random number. This procedure is repeated until the available reservoir of accretable mass is exhausted.

### Appendix A.3. Fragmentation Process

After the accretion step, agents are tested for fragmentation according to the probability defined in Equation (7). For each agent, a random number is generated and compared with its corresponding fragmentation probability. If the generated value is smaller than the assigned probability, a fragmentation event occurs.

When fragmentation takes place, the original agent is removed from the population and its mass $m_{i}$ is redistributed into $m_{i}$ unit-mass accretion centers. These newly created agents are incorporated into the population and participate in subsequent accretion events in the same manner as all other agents.

This procedure conserves the total mass of the system while increasing the number of accretion centers. Consequently, fragmentation acts as a mechanism that counterbalances the preferential growth produced by the accretion process, continuously reintroducing low-mass agents into the population and influencing the resulting mass distribution.

### Appendix A.4. Simulation Cycle and Stopping Criterion

Figure A1 summarizes the complete simulation workflow. After the initialization stage, accretion and fragmentation are evaluated sequentially at each iteration. The process continues until the accretable reservoir is exhausted, at which point the simulation terminates.

### Appendix A.5. Simplified Model Used for Parameter Exploration

In Section 5.2, an additional numerical procedure was applied to identify a suitable range of values for the parameter (β) before performing the full simulations. To isolate the influence of the fragmentation probability and reduce the computational complexity of the experiment, a simplified implementation of the model was adopted.

In this exploratory configuration, the mass contained within the accretion centers and the mass remaining in the accretable reservoir were treated as components of a single mass budget. This allowed the evolution of the fraction of material incorporated into accretion centers to be monitored throughout the simulation and provided a direct measure of the ability of the agents to undergo runaway growth.

To facilitate this analysis, in this test, fragmentation events did not create new accretion centers. Instead, a unit of mass was removed from the selected agent and returned to the mass reservoir. This modification prevented the continuous growth of the agent population and made it possible to evaluate the influence of (β) on the balance between already-accreted and still-available material, independently of the population dynamics associated with the full fragmentation process.

The complete fragmentation algorithm described in Appendix A.3 is employed in all subsequent simulations and constitutes the implementation used to generate the results presented in Section 5.3.

## Appendix B. Physical Motivation of the Model

### Appendix B.1. Jeans Instability and Characteristic Fragmentation Scales

The fragmentation prescription given by Equation (7) does not explicitly incorporate the Jeans length or Jeans mass into its dynamical rules. Nevertheless, these concepts provide the physical motivation for considering fragmentation as a fundamental process in the evolution of molecular clouds.

The Jeans mass defines a characteristic scale above which self-gravitating regions become unstable and may collapse to form denser structures. Observations and numerical simulations indicate that molecular clouds undergo hierarchical fragmentation, producing progressively smaller overdensities that may subsequently evolve into protostellar objects. In this context, fragmentation acts as a mechanism that redistributes mass among a growing population of accretion centers.

Our agent-based model does not attempt to reproduce the detailed hydrodynamics of this process, nor does it explicitly evaluate the Jeans stability of individual accretion centres. Instead, it adopts a phenomenological fragmentation prescription that is qualitatively inspired by the expectation that more massive self-gravitating structures are generally more susceptible to hierarchical fragmentation, particularly during the approximately isothermal stages of collapse.

### Appendix B.2. Accretion and Competitive Growth

The accretion rule adopted in Equation (6) is inspired by the Bondi–Hoyle–Lyttleton (BHL) accretion rate, in which the accretion rate is proportional to the square of the protostellar object’s mass. However, the BHL accretion rate considers only the central protostellar object’s mass and neglects the gaseous mass of the core where the object forms. To allow for deviations introduced by this additional mass dependence, we have treated the exponent of the mass in the accretion law as a tunable parameter that can be adjusted to recover the Salpeter power-law shape together with its observed slope.
